# Simulating the Residual Layer Thickness in Roll-to-Plate Nanoimprinting with Tensioned Webs

**DOI:** 10.3390/mi13030461

**Published:** 2022-03-18

**Authors:** Jelle Snieder, Marc Dielen, Ron A. J. van Ostayen

**Affiliations:** 1 Department of Precision and Microsystems Engineering, Delft University of Technology, Mekelweg 2, 2628 CD Delft, The Netherlands; r.a.j.vanostayen@tudelft.nl; 2 Morphotonics B.V., De Run 4281, 5503 LM Veldhoven, The Netherlands; marc.dielen@morphotonics.com

**Keywords:** nanoimprinting, roll-to-plate, residual layer thickness, simulation, elastohydrodynamic lubrication, web tension, experimental validation

## Abstract

Roll-to-plate nanoimprinting with flexible stamps is a fabrication method to pattern large-area substrates with micro- and nanotextures. The imprint consists of the preferred texture on top of a residual layer, of which the thickness and uniformity is critical for many applications. In this work, a numerical model is developed to predict the residual layer thickness (RLT) as a function of the imprint parameters. The model is based on elastohydrodynamic lubrication (EHL) theory, which combines lubrication theory for the pressure build-up in the resin film, with linear elasticity theory for the elastic deformation of the roller material. The model is extended with inextensible cylindrical shell theory to capture the effect of the flexible stamp, which is treated as a tensioned web. The results show that an increase in the tension of the web increases the effective stiffness of the roller, resulting in a reduction in the RLT. The numerical results are validated with layer height measurements from flat layer imprints. It is shown that the simulated minimum layer height corresponds very well with the experimental results for a wide range of resin viscosities, imprint velocities, and imprint loads.

## 1. Introduction

Nanoimprint lithography (NIL) is a high-resolution, high-throughput fabrication technology to replicate micro- and nanopatterns on rigid and flexible substrates. The concept of NIL is essentially based on a moulding process, in which a liquid resist is deformed to conform to the surface topography of a stamp [1]. The stamp surface is equipped with the inverse polarity of the preferred texture. After solidification of the resist material, the stamp is removed and a negative of the pattern is replicated onto the substrate. A distinction can be made between thermal NIL with a thermoplastic polymer resist material [2], and ultraviolet (UV) cure NIL, which employs UV-curable resin [3]. Moreover, depending on the contact method, the NIL process can be divided into plate-to-plate, roll-to-plate, and roll-to-roll nanoimprinting. To further increase the throughput and imprint area of the NIL process, a shift towards large-area NIL can be identified [4]. Practical applications of large-area NIL can be found in the fabrication of optical films for displays or augmented reality [5], anti-reflection surfaces for solar panels [6], antifouling surfaces [7], and many more [4]. In particular, roller-based NIL is an attractive method, as it offers the advantage of a reduced contact area during the imprint process, which results in lower imprint forces, and reduced issues regarding bubble trapping [8]. The first thermal NIL roller-based imprint system was introduced by Tan [9], and Ahn has developed the first UV-cure roll-to-roll imprint system [10]. Various roller-based imprint systems are available, and can be classified based on the configuration of stamp, roller, and substrate [11,12].

**Figure 1:**
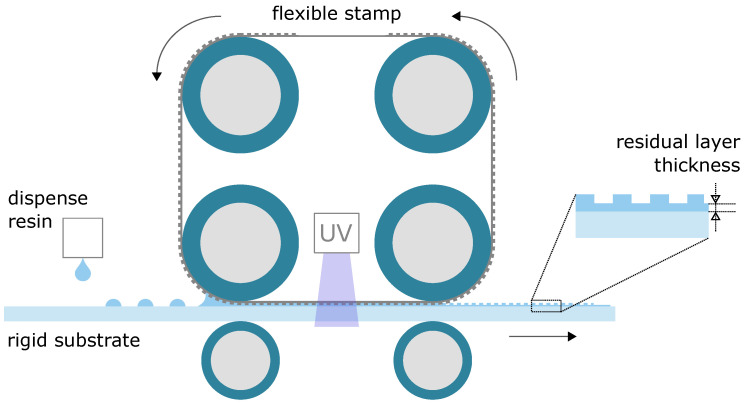
Schematic of a roll-to-plate imprint system. Picture is adapted from [20].

A roll-to-plate imprint system can be equipped with one or multiple rollers. Figure 1 shows a schematic of a UV-cure roll-to-plate imprint system. It uses multiple rollers, for imprinting and for guiding a textured flexible stamp. The UV-curable resin is dispensed on the substrate. The middle-left imprint roller presses the flexible stamp into the liquid resin, which is then UV-cured through the transparent stamp. The middle-right roller delaminates the flexible stamp from the hardened resin. When taking a closer look at the imprinted substrate, it can be seen that the imprint consists of the preferred texture on top of a residual layer. For many applications, the residual layer thickness (RLT) must be thin and uniform, in order to prevent poor optical or mechanical performance of the product, or to facilitate efficient and high-quality etching as a subsequent process step to transfer the pattern into the substrate material [13]. Simulation of the imprint process can assist in predicting the imprint quality to further optimize the imprint process. The imprint quality is mainly governed by the replication fidelity of the preferred textures and the RLT and its uniformity over the imprint area. In the literature, research on the simulation of the replication fidelity can be found for different aspects of the imprint process: the texture filling and potential bubble trapping [14,15,16,17], the UV curing step [18,19], and the potential fracture of textures during delamination [16], to name a few. This work specifically focuses on the prediction of the RLT.

Previous work on the simulation of the RLT and its uniformity is given for plate-to-plate imprint systems [21,22,23,24]. For roller-based imprint systems, analytical expressions for the RLT [25] and droplet merging under the imprint roller [18] are presented. The elastic deformation of the roller material is not taken into account. It is included by Taylor [26], who developed a contact mechanics-based model to simulate the RLT. The rollers in roller-based imprint systems are typically equipped with a relatively soft, elastomeric layer, which elastically deforms during the imprint process, due to the hydrodynamic pressure build-up in the resin film. The elastic deformation is relatively large compared to the film height, and affects the hydrodynamic pressure profile in the resin film, in return. The result is a strong coupling between the hydrodynamic and elastic effects, which is described by elastohydrodynamic lubrication (EHL) theory. The working principle behind roller-based imprint systems is analogous to roller-based coating and printing processes [27,28,29,30]. These studies also use EHL theory for the prediction of the coating or printing layer height. When the thickness of the elastomeric layer is relatively small, it influences the elastic deformation and its finite thickness needs to be taken into account [28,31,32,33]. Cochrane also employs EHL theory to predict the RLT in a roll-to-roll set-up to imprint deformable substrates [34]. Lubrication theory is combined with the inextensible cylindrical shell theory to couple the pressure build-up in the thin film of resin with the motion and elastic forces of the deformable substrate.

In this work, a numerical EHL model is developed to predict the RLT in roll-to-plate imprint systems. The EHL model couples lubrication theory for the pressure build-up in the thin film of resin with linear elasticity theory for the elastic deformation in the elastomeric layer of the imprint roller with finite thickness. The model is extended with inextensible cylindrical shell theory to include the effect of the flexible stamp, which is treated as a tensioned web. The study focuses on the RLT, and the influence of textures on the flexible stamp is not taken into account. Unique contributions of this work are the implementation of EHL theory in roll-to-plate nanoimprinting, including the experimental validation of the numerical results. Moreover, the effects of the tensioned web in a roll-to-plate imprint set-up on the RLT have not been studied before, to the authors’ knowledge. The numerical model can directly be used to determine the required amount of resin in an imprint, for a given set of imprint parameters. Furthermore, the numerical model can be used to study the RLT as a function of the resin viscosity, imprint velocity, and imprint load. The material properties of the elastomeric layer (elastic modulus and Poisson ratio) and the system geometry (roller radius and elastomeric layer thickness) can be adjusted as well, in order to study the influence on the RLT.

## 2. Methods

In this section, the applied methods are described in detail. First, the development of the numerical model for the lubricated roller contact with a tensioned web is discussed. Next, the experimental method to validate the model is described.

### 2.1. Model Development

**Figure 2:**
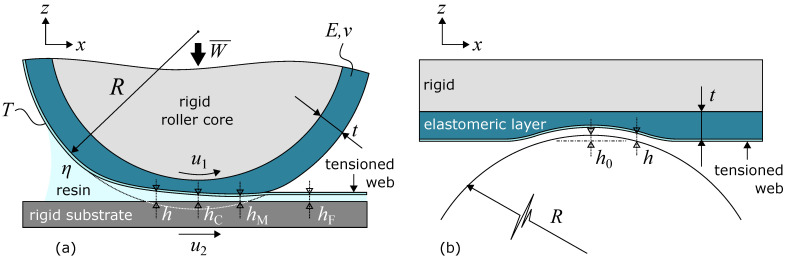
(**a**) Schematic of the imprint roller with tensioned web. The elastic deformation of the elastomeric layer is highly exaggerated for illustrative purposes. (**b**) Equivalent geometry of the imprint roller with tensioned web.

The numerical model is based on the full-system finite element approach for EHL problems [35]. The modeled system geometry is shown in Figure 2a. The imprint roller with an elastomeric layer is pressed onto the substrate. They are separated by a tensioned web, which applies a contact pressure onto the elastomeric layer, and a thin film of resin. The roller contact is treated as an infinite line contact. Furthermore, it is assumed that the substrate and roller core are rigid, and all elastic deformation occurs in the relatively compliant, elastomeric layer, which is wrapped around the imprint roller core. If the contact width is small relative to the roller radius, a simplified equivalent geometry can be used, as shown in Figure 2b [36]. A rigid roller of radius *R* is pressed onto the flat, elastomeric layer of thickness *t*, which is essentially unwrapped from the roller core. With this implementation, the tensioned web follows the roller shape, instead of losing contact with the roller and moving away with the substrate. The kinematics of the tensioned web and its contact mechanics with the roller are not taken into account. Both the roller and substrate move with a unidirectional surface velocity $u_{1}$ and $u_{2}$, in the positive *x*-direction. This is in fact the imprint velocity of the imprint process. The contact area is subject to an effective imprint load per unit length $\bar{W}$. It is assumed that a surplus of resin is available in front of the roller, which makes the contact fully flooded.

Three layer heights are identified in the formed thin film of resin: the central layer height $h_{C}$ at the roller center, the minimum layer height $h_{M}$ close to the outlet of the roller contact, and the final layer height $h_{F}$ between the tensioned web and substrate. The final layer height $h_{F}$ is the layer height of interest, but due to the absence of the web kinematics, this layer height cannot be determined directly. However, it will be shown in Section 2.1.2 that the final layer height $h_{F}$ should be equal to the central layer height $h_{C}$.

**Figure 3:**
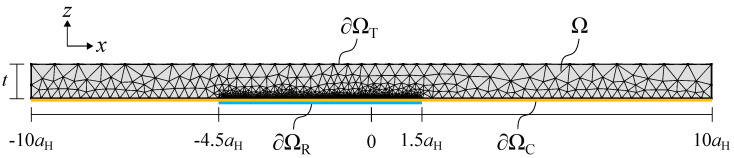
Computational domain and mesh.

The numerical model is governed by four main equations: the Reynolds equation for the resin flow, the linear elasticity equations for the elastic deformation, the inextensible cylindrical shell equations for the web tension physics, and a load balance equation. For the sake of numerical robustness and faster convergence, the relevant variables are scaled. The scaling parameters and dimensionless equations are presented in the Supplementary Materials. For readability, the dimensional equations are presented in the following subsections. The equations are applied on the computational domain in Figure 3, which represents the elastomeric layer. It has a dimensional width of $20\times a_{H}$, and a dimensional height which is equal to the elastomeric layer thickness. The parameters $a_{H}$ and $p_{H}$, which are already introduced for later use, correspond to the Hertz dry contact half-width and peak pressure, respectively. They are based on the mechanical properties of the elastomeric layer:

(1) $$a_{H}=\sqrt{\frac{8\bar{W}R}{\pi E^{\prime }}},\;p_{H}=\frac{2\bar{W}}{\pi a_{H}}.$$

As it is assumed that all elastic deformation occurs in the elastomeric layer, the effective elastic modulus reduces to:

(2) $$\frac{2}{E^{\prime }}=\frac{1-\nu^{2}}{E}.$$

#### 2.1.1. Elastic Deformation

The elastic deformation is determined by applying the classical linear elasticity equations on the elastic layer domain Ω in Figure 3, with appropriate boundary conditions. The linear elasticity equations are given by [35]:

(3) $$\begin{array}{c} \begin{array}{cc} x-\mathrm{direction}:\; & \frac{\partial }{\partial x}\left[\left(\lambda +2\mu \right)\frac{\partial u}{\partial x}+\lambda \frac{\partial w}{\partial z}\right]+\frac{\partial }{\partial z}\left[\mu \left(\frac{\partial u}{\partial z}+\frac{\partial w}{\partial x}\right)\right]=0,\\ z-\mathrm{direction}:\; & \frac{\partial }{\partial x}\left[\mu \left(\frac{\partial u}{\partial z}+\frac{\partial w}{\partial x}\right)\right]+\frac{\partial }{\partial z}\left[\lambda \frac{\partial u}{\partial x}+\left(\lambda +2\mu \right)\frac{\partial w}{\partial z}\right]=0. \end{array} \end{array}$$

where λ and μ correspond to the Lamé parameters:

(4) $$\lambda =\frac{\nu E}{\left(1-2\nu \right)\left(1+\nu \right)},\;\mu =\frac{E}{2\left(1+\nu \right)}.$$

The upper boundary $\partial \Omega_{T}$ in Figure 3 is fixed. The contact domain $\partial \Omega_{c}$ is loaded with the contact pressure $p_{C}$, which follows from the tensioned web physics. This results in the following boundary conditions:

(5) $$\left\{\begin{array}{cc} u=w=0\; & \mathrm{on}\;\partial \Omega_{T},\\ \sigma_{n}=p_{C}\; & \mathrm{on}\;\Omega_{C},\\ \sigma_{n}=\sigma_{t}=0\; & \mathrm{elsewhere}. \end{array}\right.$$

The parameters $\sigma_{n}$ and $\sigma_{t}$ are the normal and tangential components of the stress tensor, respectively.

#### 2.1.2. Hydrodynamic Lubrication

As the layer thickness of the thin film of resin is small compared to the roller contact width, the resin flow can be described by thin film theory, which assumes a constant pressure across the film thickness. The steady-state, incompressible Reynolds equation in one dimension is given by [37]:

(6) $$\frac{\partial }{\partial x}\left(-\frac{h^{3}}{12\eta }\frac{\partial p}{\partial x}+\frac{h\left(u_{1}+u_{2}\right)}{2}\right)=0,$$

where η is the dynamic viscosity of the resin, and $u_{1}$ and $u_{2}$ are the top and bottom surface velocities, respectively. Equation (6) assumes Newtonian fluid behavior and isothermal conditions. The first part in the Reynolds equation is the Poiseuille term, which describes the volume flow rate due to pressure gradients within the thin film. The second term is the Couette term and describes the volume flow rate due to the surface velocities. The hydrodynamic film pressure *p* is determined for a given layer height profile *h*:

(7) $$h\left(x\right)=h_{0}+\frac{x^{2}}{2R}+w\left(x\right),$$

where $h_{0}$ is the unknown gap between the roller and substrate at x=0. A negative gap indicates roller engagement, as shown in Figure 2b. The second term is an approximation to describe the circular roller shape, and the last term represents the elastic deformation, which follows from Equation (3). The Reynolds equation is applied on domain $\partial \Omega_{R}$ in Figure 3. It is defined by $-4.5a_{H}\leq x\leq 1.5a_{H}$, which is sufficiently wide to capture the pressure build-up in the thin film of resin [35]. Zero pressure boundary conditions are applied on the edges of the domain. In the outlet region of the roller contact, negative pressures will follow from the Reynolds equation, due to the diverging surfaces. These negative pressures are physically not tolerated and the fluid will cavitate. The location of the cavitation boundary is unknown beforehand. When it is assumed that the cavitation pressure is equal to ambient pressure, the following cavitation condition must be satisfied:

(8) $$p\geq 0\;\mathrm{on}\;\partial \Omega_{R},\;\mathrm{and}\;p=\frac{\partial p}{\partial x}=0\;\mathrm{on}\;\mathrm{the}\;\mathrm{cavitation}\;\mathrm{boundary}.$$

To satisfy the cavitation condition, different methods are available and implemented in the literature. Habchi [38] uses a penalty method to force any negative pressures towards zero. Other methods are based on the observation that two regions can be identified: the full film region in which *p* is unknown (but larger than 0) and the liquid volume fraction *f* is known (namely 1), and the cavitated region where *p* is known (namely 0) and the liquid volume fraction *f* is unknown (but smaller than 1). This reasoning can be captured in a complementarity condition when introducing the cavity fraction θ=1−f:

(9) $$\begin{array}{c} \begin{array}{cc} p\geq 0\; & \mathrm{and}\;\theta =0,\\ p=0\; & \mathrm{and}\;\theta \geq 0, \end{array} \end{array}$$

Alakhramsing [39] suggests a variable transformation to combine both *p* and *f* into one variable. In this work, the complementarity condition is satisfied by adding a modified constraint function to the model, known as the Fischer–Burmeister function [40]:

(10) $$p+\theta -\sqrt{p^{2}+\theta^{2}}=0.$$

The Reynolds equation in Equation (6) is essentially a mass flow balance, and conservation of mass must be satisfied on the entire domain. Similar conditions are present at the roller center (at x=0) and outlet (at the cavitation boundary). The pressure gradient ∂p/∂x is equal to zero and the surface velocities $u_{1}$ and $u_{2}$ are the same. Conservation of mass then yields that the layer height at the cavitation boundary should be equal to the central layer height $h_{C}$ at the roller center. As the layer height is assumed to be constant downstream of the roller outlet, the final layer height $h_{F}$ should be equal to the central layer height $h_{C}$.

#### 2.1.3. Web Tension

The tensioned web is relatively thin, and therefore cylindrical shell equations can be used to describe the web tension physics [34,41]. It is assumed that the bending stiffness of the web and tangential traction acting on the web are negligible. The normal stress balance reduces to:

(11) $$\kappa T+p_{n}=0.$$

This equation states that the normal stress or pressure $p_{n}$ is a function of the web curvature κ and the applied web tension *T*. The tensioned web follows the roller shape and its potential elastic deformation, which are described by the last two terms in Equation (7), respectively. The curvature can be approximated by the second spatial derivative:

(12) $$\kappa =-\frac{\partial^{2}z_{\mathrm{roller}}}{\partial x^{2}}=-\frac{\partial^{2}}{\partial x^{2}}\left(\frac{x^{2}}{2R}+w\right)=-\left(\frac{1}{R}+\frac{\partial^{2}w}{\partial x^{2}}\right).$$

The normal stress is equal to the tensioned web contact pressure $p_{C}$ minus the hydrodynamic film pressure *p*. The resulting normal stress balance is equal to:

(13) $$p_{C}=p+T\left(\frac{1}{R}+\frac{\partial^{2}w}{\partial x^{2}}\right).$$

Equation (13) explains that the web contact pressure, which acts on the elastomeric layer, is the sum of the hydrodynamic film pressure and the pressure as induced by the web tension itself. This last term scales with the curvature of the roller and the local curvature due to the elastic deformation of the elastomeric layer. Equation (13) is applied on the contact domain $\partial \Omega_{C}$ in Figure 3. Zero pressure boundary conditions for the web tension pressure are applied on the edges of the domain.

#### 2.1.4. Load Balance

The load equilibrium is derived by balancing the pressure force from the hydrodynamic film pressure build-up with the effective roller load per unit length:

(14) $$\int_{\Omega_{R}}pdx=\bar{W}.$$

This equation is satisfied by regulating the gap $h_{0}$ in Equation (7), which is one of the unknowns in the system of equations.

#### 2.1.5. Numerical Implementation

The linear elasticity equations in Equation (3), the Reynolds equation in Equation (6), the Fischer–Burmeister function in Equation (10), the tensioned web equation in Equation (13), and the load balance equation in Equation (14) completely define the EHL model with a tensioned web. The unknowns of these equations are the elastic deformation components *u* and *w*, the hydrodynamic film pressure *p*, the cavity fraction θ, the tensioned web contact pressure $p_{C}$, and the constant film thickness gap $h_{0}$. The equations are implemented in the commercial finite element method (FEM) software COMSOL Multiphysics^®^ [42]. The Reynolds equation in Equation (6) and web tension equation in Equation (13) are discretized using second-order (quadratic) Lagrangian finite elements. The linear elasticity equations in Equation (3) are discretized using third-order (cubic) Lagrangian finite elements, to allow for a smooth second-order derivative of the curvature in Equation (13). The load balance equation is a simple ordinary integral equation, which is associated with the unknown constant film thickness gap $h_{0}$. It is directly added to the system of equations as formed by Equations (3), (6) and (13), together with the Fischer–Burmeister constraint function.

The Reynolds equation, which is an example of a typical convection–diffusion equation, is convection-dominated in the cavitated region, where the film pressure is equal to zero. Convection-dominated partial differential equations are known to be unstable using FEM [35]. Therefore, the formulation is stabilized with a mesh-dependent artificial diffusion term. Moreover, to speed up the solution time, a small mesh-dependent diffusion term is added to the Fischer–Burmeister equation.

The use of FEM allows for non-structured, non-regular meshing of the computational domain; see Figure 3. A relatively course triangular mesh is used in the solid domain. The mesh is refined at the lower boundaries $\partial \Omega_{C}$ and $\partial \Omega_{R}$. The mesh is set fine in the inlet and outlet regions ($-4.5a_{H}\leq x\leq -a_{H}$ and $a_{H}\leq x\leq 1.5a_{H}$), finer in the central (Hertz) roller contact zone ($-a_{H}\leq x\leq 0.5a_{H}$), and finest in the outlet of the central contact zone ($0.5a_{H}\leq x\leq a_{H}$), where the pressure and elastic deformation gradients are most important. Mesh convergence studies have been performed to guarantee a mesh-independent solution. The solution procedure starts with selecting an appropriate initial guess for the unknown *p*, $p_{C}$, *u*, *w*, and $h_{0}$. The Hertz dry contact pressure is taken as an initial guess for the Reynolds pressure and tensioned web contact pressure. It is defined by:

(15) $$p_{R},\mathrm{initial}=p_{C},\mathrm{initial}=\left\{\begin{array}{cc} p_{H}\sqrt{\left(1-\frac{x^{2}}{a_{H}^{2}}\right)} & \mathrm{for}\;-a_{H}\leq x\leq a_{H},\\ 0 & \mathrm{elsewhere}. \end{array}\right.$$

The elastic deformation resulting from the Hertz dry contact pressure is taken as an initial guess for the elastic deformation components *u* and *w*. The initial guess for the constant film thickness gap $h_{0}$ is simply taken as a small, positive number (e.g., 10 μm). The set of equations is iteratively solved in a fully coupled manner using a damped Newton–Raphson approach until convergence is reached.

### 2.2. Experimental

**Figure 4:**
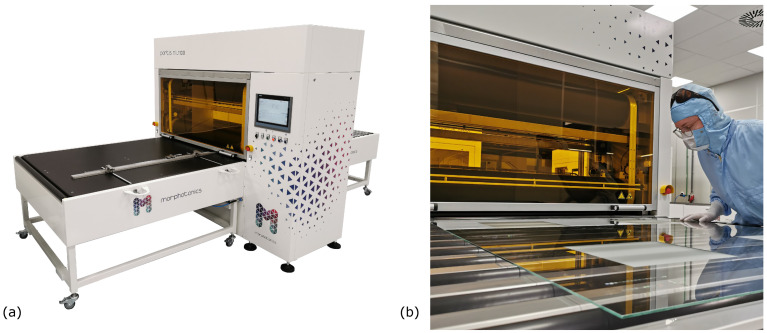
(**a**) Morphotonics Portis NIL1100 roll-to-plate nanoimprint equipment [20]. (**b**) Detailed view of the rollers inside the Morphotonics Portis NIL1100 nanoimprint tool.

The numerical EHL model is validated with experimental results. Multiple flat layer imprints have been made on a Morphotonics Portis NIL1100 roll-to-plate nanoimprint set-up [20]; see Figure 4. A schematic of the working principle is shown in Figure 1. Each imprint is performed on a flat 150 mm×150mm glass substrate of 0.5mm thickness, which is placed on a thick 5 mm glass carrier plate. To improve the adhesion between the resin and the glass substrates, the substrates are cleaned with isopropyl alcohol and pretreated with atmospheric pressure oxygen plasma and a primer containing an adhesion promoter. The flat layer imprints are fabricated using a flexible polymer stamp without textures. The stamp is pre-tensioned around the test rollers, which are equipped with a 7.5mm thick elastomeric layer with an elastic modulus of 3.2MPa and a Poisson ratio of 0.47. The linear elastic material behavior is confirmed by experimental compression tests.

The experimental validation procedure consists of multiple measurement series, in which the resin viscosity, imprint velocity, and imprint load are varied. Five different in-house-developed acrylate-based resins are used [20]. The viscosities and volumetric shrinkage levels upon UV curing are listed in Table 1. The viscosity is determined at 25 °C.

The imprint layer height of each imprint is determined using a Keyence VK-X1100 laser confocal microscope, by optically measuring the step height of a small scratch made in the cured imprint layer. This is done on multiple locations of the imprint surface, to determine the layer height average and variation. To be able to compare the modeled layer heights with the measured, cured layer heights, the latter are converted to the liquid, pre-cured layer height using the shrinkage values in Table 1. Finally, the material properties of the elastomeric layer and resin are considered to be the most sensitive to variations in environmental temperature or material composition. A variation of ±10% in both the elastic modulus of the elastomeric layer and the resin viscosity is included in the model, to provide insight into their impact on the layer height. The amount of variation is based on measurements of the elastic modulus of the elastomeric layer and the resin viscosity, given the possible variations in the material composition of the elastomeric layer and the temperature dependency of the viscosity.

## 3. Results

This section presents the numerical and experimental results. The numerical results give an indication of the pressure and layer height profiles within the roller contact, including the influence of web tension. Next, the numerical results are validated with experimental results for a wide range of imprint parameters.

### 3.1. Model

**Figure 5:**
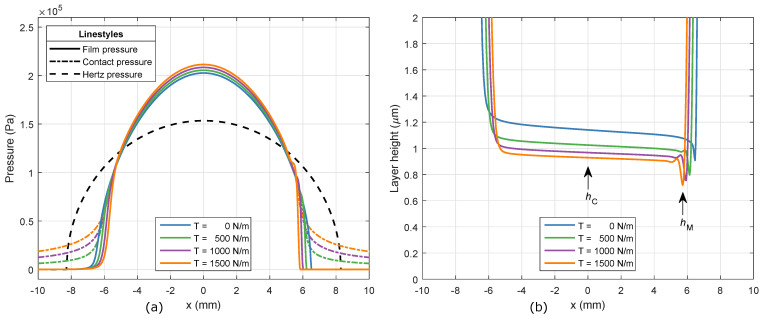
(**a**) The hydrodynamic film pressure $\left(p\right)$ and tensioned web contact pressure $\left(p_{C}\right)$ along the *x*-coordinate for varying web tension values. The Hertz dry contact pressure profile from Equation (15) is shown for reference. (**b**) The layer height $\left(h\right)$ along the *x*-coordinate for varying web tension values.

The numerical model has been run with varying web tension values and a specific set of imprint parameters: a resin viscosity of 30 mPa s, an imprint velocity of 3 mm/s^−1^, and an imprint load of 2000 N m^−1^. The resulting pressure profiles for the hydrodynamic film pressure and tensioned web contact pressure are shown in Figure 5a. It also shows the Hertz dry contact pressure, for reference. The Hertz pressure and contact half-width are equal to $1.53\times 10^{5}$ Pa and 8.3mm, respectively. Starting from the inlet of the roller contact, the hydrodynamic film pressure smoothly increases up to the peak pressure in the center $\left(x=0\right)$, after which it decreases to ambient pressure again. The finite thickness of the elastomeric layer results in smaller contact widths and larger peak pressures in the resin film, compared to the Hertz solution. A similar phenomenon can be identified when web tension is included in the model. The tensioned web restricts the elastic deformation of the elastomeric layer material, thereby increasing the effective stiffness of the roller contact. For zero web tension, the tensioned web contact pressure is equal to the hydrodynamic film pressure, as also indicated by Equation (13). Increasing values of the web tension result in smaller contact widths and increased peak pressures in the thin film of resin. Outside the roller contact zone, the tensioned web contact pressure approaches a constant value of T/R. This can be explained by an absence of hydrodynamic film pressure, while the second-order derivative of the elastic deformation in Equation (13) approaches zero.

The corresponding layer height profiles are shown in Figure 5b. Due to the diverging surface of the roller, the layer height rapidly decreases until the roller contact zone. Within the contact zone, the layer height follows a nearly uniform, slowly decreasing profile. The central layer height $h_{C}$ in the center and the minimum layer height $h_{M}$ near the outlet are clearly visible. This does not hold for the final layer height $h_{F}$. With the implemented model approach, the tensioned web follows the roller shape, which quickly increases after the location of the minimum layer height. For zero web tension, the central and minimum layer heights are equal to 1.14 μm and 0.91 μm, respectively. The influence of web tension is in line with the effect on the pressure profiles. The layer height decreases for increasing web tension, due to the increased effective stiffness of the roller contact.

### 3.2. Experimental Validation

**Figure 6:**
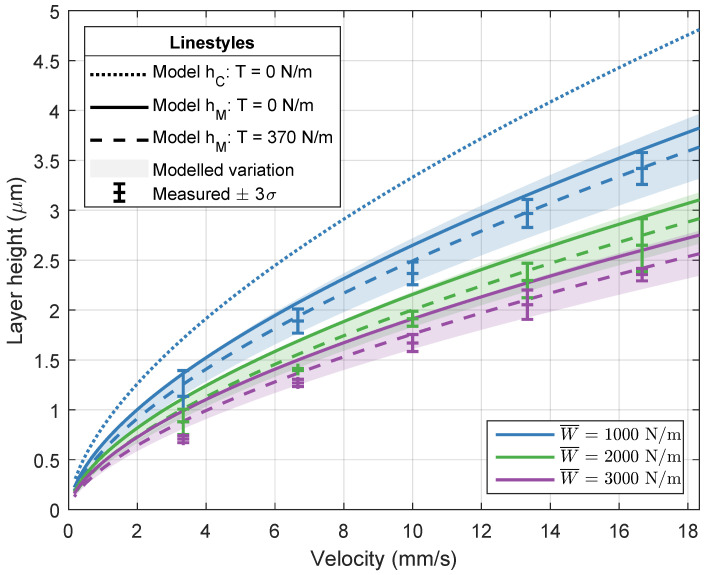
Numerical and experimental results for the layer height for varying imprint loads and imprint velocities. The modeled results include a ±10% variation in both elastic modulus of the elastomeric layer and resin viscosity. The simulations and imprints are performed with Resin B from Table 1 (viscosity of 38 mPa s).

**Figure 7:**
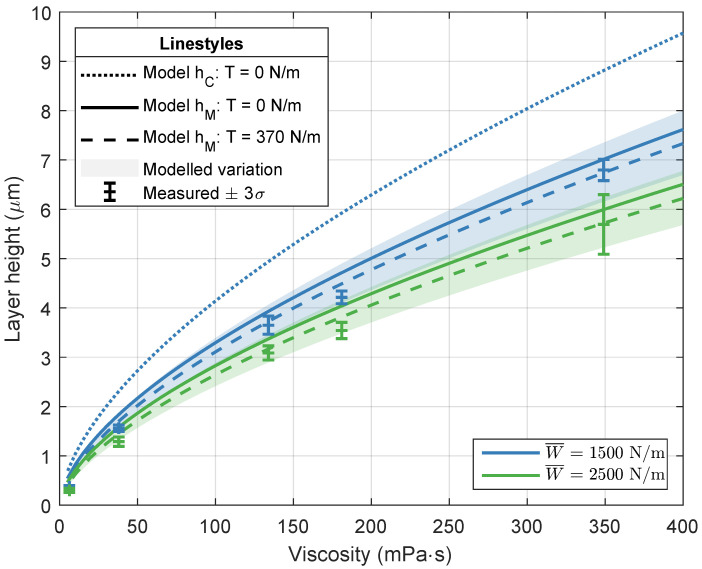
Numerical and experimental results for the layer height for varying imprint loads and resin viscosities. The modeled results include a ±10% variation in both elastic modulus of the elastomeric layer and resin viscosity. The simulations and imprints are performed with a constant imprint velocity of 6.7 mm s^−1^. The imprints are performed with the resins as listed in Table 1.

**Figure 8:**
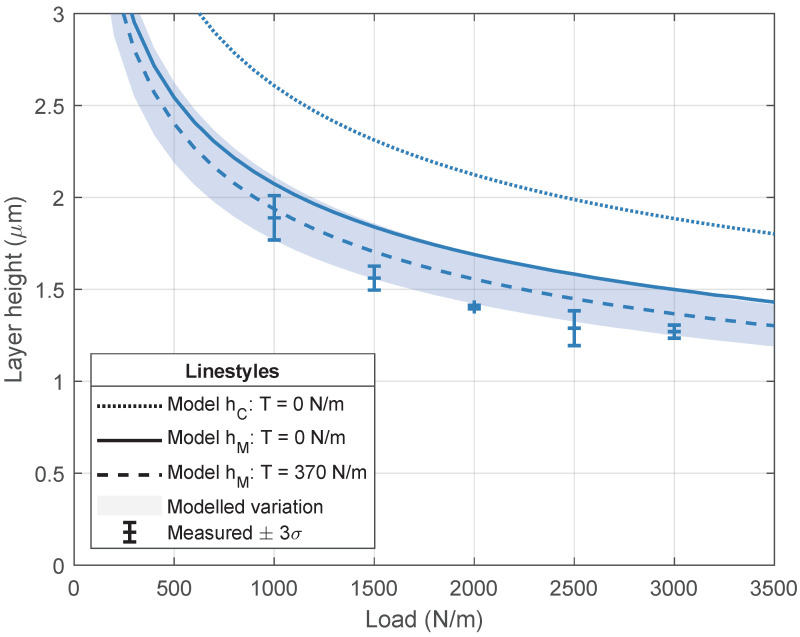
Numerical and experimental results for the layer height for a varying imprint load. The modeled results include a ±10% variation in both elastic modulus of the elastomeric layer and resin viscosity. The simulations and imprints are performed with Resin B from Table 1 (viscosity of 38 mPa s) and a constant imprint velocity of 6.7 mm s^−1^.

Figure 6, Figure 7 and Figure 8 visualize the simulated and measured layer heights for different imprint loads and for a variation in imprint velocity, resin viscosity, and imprint load, respectively. The modeled variation of ±10% in both the elastic modulus of the elastomeric layer and the resin viscosity is shown as well. The variation is modeled around the results including web tension. The results in each graph will be discussed separately.

Figure 6 visualizes the layer heights for varying velocity and three different imprint loads. In both model and measurement, the viscosity is kept constant at 38 mPa s. The layer height increases with increasing velocity and decreasing load. Furthermore, the layer height slightly decreases when the web tension of 370 N m^−1^ is taken into account. This behavior is also shown in Figure 5b. When taking a closer look at the layer height for a 1000 N m^−1^ imprint load, it can be seen that there is good agreement between the measured layer heights and the minimum layer height from the numerical model with web tension. Contrary to the hypothesis, the minimum layer thickness seems to be the best predictor of the RLT, instead of the central layer thickness. For clarity, the central layer heights for the other imprint loads, which show similar behavior, are not shown. The layer heights for a varying resin viscosity and two different imprint loads are shown in Figure 7. The imprint velocity is kept constant at 6.7 mm s^−1^. The results are comparable to the results for a varying imprint velocity in Figure 6. The layer height increases with increasing resin viscosity and decreasing imprint load. Again, good agreement is found between the measured layer heights and the minimum layer height from the numerical model. The layer height for a varying imprint load is shown in Figure 8. The experimental data in this graph are in fact deduced from the measurements in Figure 6 and Figure 7. The resin viscosity and imprint velocity are kept constant at 38 mPa s and 6.7 mm s^−1^, respectively. The layer height decreases with increasing imprint load, as expected. Again, good agreement is found between the measured layer heights and the minimum layer height from the numerical model.

## 4. Discussion

The numerical results for the minimum layer thickness and the experimental results agree very well. This contradicts the hypothesis that the final layer thickness corresponds to the central layer thickness. The validity of the numerical model and the corresponding results will be discussed.

The EHL model consists of different physics, each with its own assumptions. The assumptions in material properties and behavior are considered to be the most critical. The relevant materials in the imprint process are the elastomeric layer and the imprint resin. The numerical results in Figure 6, Figure 7 and Figure 8 include a modeled variation of ±10% in both the elastic modulus of the elastomeric layer and the viscosity of the resin. The results clearly indicate that any changes in the material properties have a direct impact on the layer height of the imprint. The elastic deformation in the elastomeric layer is described by linear elasticity theory, which assumes small deformations and a linear relation between stress and strain. Although the elastic deformation is large compared to the layer height, it is still small compared to the elastomeric layer thickness. For the maximum load case of 3000 N m^−1^, the maximum elastic deformation is equal to 0.26 mm. This corresponds to a linear strain of 0.034, which is considered to be small. The resin is the other relevant material in the imprint process. It is assumed to be isoviscous. In practice, the resin viscosity can depend on pressure, shear rate, and temperature. The EHL contact is part of the soft EHL regime, which is characterized by relatively low contact pressures [43,44], as can also be seen in the typical film pressure profiles in Figure 5a. This confirms the assumption that any piezoviscous effects can be neglected. The Newtonian fluid behavior, which assumes a shear-rate-independent viscosity, is confirmed by viscosity measurements for a varying shear rate. Moreover, because the roller and substrate move with a similar velocity, the shear rate in the thin film of resin will be relatively low. Lastly, the process is assumed to be isothermal. It is known that the resin viscosity depends on temperature, similar as with other fluids and lubricants [37]. However, because the location of curing is relatively far way from the imprint roller (see Figure 1), any heating due to the UV source or the exothermal curing process can be neglected. This is confirmed by monitoring the imprint roller temperature during the experiments.

An important difference between the numerical model and the experimental set-up is the contact mechanics and kinematics of the tensioned web. The numerical model assumes that the tensioned web follows the imprint roller, as shown in Figure 2b and Figure 5b. In reality, the tensioned web and roller lose contact in the outlet region, as the tensioned web moves away with the substrate in the imprint direction and the contact pressure diminishes. Furthermore, the bending stiffness of the tensioned web is not yet taken into account. The tensioned web is a relatively flexible stamp. The bending stiffness will be low, but might not be negligible. The hypothesis is that the bending stiffness slightly increases the effective stiffness of the roller contact, which results in a small reduction in the RLT. It is believed that both aspects must be included to better describe the roll-to-plate imprint process with tensioned, flexible stamps.

## 5. Conclusions

In this work, a numerical model is developed to predict the imprint layer thickness in UV-cure roll-to-plate nanoimprinting. The numerical model combines multiple physics in an elastohydrodynamic lubrication model to describe the fluid flow of the thin film of resin, the elastic deformation of the elastomeric layer, the mechanics of the tensioned web, and the coupling between them. We have shown that the simulated minimum film thickness in the roller contact corresponds very well to the experimental layer thickness values for a wide range of resin viscosities, imprint velocities, and imprint loads. The model finds direct practical use for determining the required amount of resin in a specific imprint, for a given set of machine and process parameters. Furthermore, it can be employed to study the impact of the various parameters in the imprint process on the RLT and its uniformity over the imprint area. Future work will address the contact mechanics of the tensioned web and the roller, the bending stiffness and kinematics of the tensioned web, and the influence of different textures on the resin flow and the RLT. These extensions of the numerical model will help in an even better understanding of the roller-based nanoimprint process to further improve the prediction of the RLT in UV-cure roll-to-plate nanoimprinting.

## Figures and Tables

**Table 1:** Properties of the imprint resins. The viscosities are measured at 25 °C.

Resin	Viscosity (mPa s)	Volumetric Shrinkage (%)
A	6.3	12.5
B	38	8.1
C	134	7.2
D	181	8.8
E	349	7.8

## Data Availability

The data presented in this study are available on request from the corresponding author.

## Supplementary Materials

The description of the dimensionless equation set-up is available online at https://www.mdpi.com/article/10.3390/mi1010000/.

## Nomenclature

*Abbreviations*	
EHL	Elastohydrodynamic Lubrication
FEM	Finite Element Method
NIL	Nanoimprint Lithography
RLT	Residual Layer Thickness
UV	Ultraviolet
*Symbols*	
*a*_H_	Hertz contact half-width (m)
*E*	Elastic modulus (Pa)
*E*′	Effective elastic modulus (Pa)
*f*	Liquid volume fraction (-)
*h*	Film/layer height (m)
*h*_0_	Gap between roller and substrate at *x* = 0 (m)
*h*_C_	Central layer height (m)
*h*_F_	Final layer height (m)
*h*_M_	Minimum layer height (m)
*p*	Hydrodynamic film pressure (Reynolds) (Pa)
*p*_C_	Tensioned web contact pressure (Pa)
*p*_H_	Hertz contact pressure (Pa)
*p*_n_	Normal pressure on the tensioned web (Pa)
*R*	Roller radius (m)
*T*	Web tension (N m^−^^1^)
*t*	Elastomeric layer thickness (m)
*u*	Elastic deformation in *x* (m)
*u*_1_	Roller surface imprint velocity (m s^−^^1^)
*u*_2_	Substrate surface imprint velocity (m s^−^^1^)
*w*	Elastic deformation in *z* (m)
$\bar{W}$	Effective imprint load per unit length (N m^−^^1^)
*x*	Space coordinate in horizontal direction (m)
*z*	Space coordinate in vertical direction (m)
*z*_roller_	Roller height profile (m)
*η*	Resin dynamic viscosity (Pa s)
*θ*	Cavity fraction (1 − *f* ) (-)
κ	Curvature of the tensioned web (m^−^^1^)
*λ*	Lamé’s first parameter (Pa)
*μ*	Lamé’s second parameter (Pa)
*v*	Poisson ratio (-)
*σ* *_n_*	Normal component of the stress tensor (Pa)
*σ* *_t_*	Tangential component of the stress tensor (Pa)
