# Supplementary material for: Simulating the Residual Layer Thickness in Roll-to-Plate Nanoimprinting with Tensioned Webs
Source: Micromachines (Basel). 2022 Mar 18;13(3):461. doi: 10.3390/mi13030461 (PMC8955766; doi:10.3390/mi13030461)
Supplement: Supplementary file 1 [file micromachines-13-00461-s001.zip › micromachines-1636693-supplementary.pdf]

Supplementary material

Jelle Snieder[1,2,*], Marc Dielen[2] and Ron A. J. van Ostayen[1]

[1] *Department of Precision and Microsystems Engineering, Delft University of Technology, Mekelweg 2, 2628 CD, Delft, the Netherlands; r.a.j.vanostayen@tudelft.nl*
[2] *Morphotonics B.V., De Run 4281, 5503 LM Veldhoven, The Netherlands; marc.dielen@morphotonics.com*
[*] *Correspondence: j.snieder@tudelft.nl*

This document describes the dimensionless equation set-up for the elastohydrodynamic lubrication model with tensioned webs. The equation parameters are not introduced in full detail. Their explanation can be found in the main publication and the nomenclature in Appendix B.

A Dimensionless equations

The dimensionless hydrodynamic film pressure P, tensioned web contact pressure P_C, film height H, elastic deformation components U and W, and spatial coordinates X and Z are defined as follows:

$$P = \frac{p}{p_\mathrm{H}}, \quad P_\mathrm{C} = \frac{p_\mathrm{C}}{p_\mathrm{H}}, \quad H = \frac{hR}{a_\mathrm{H}^2}, \quad U = \frac{uR}{a_\mathrm{H}^2}, \quad W = \frac{wR}{a_\mathrm{H}^2}, \quad X = \frac{x}{a_\mathrm{H}}, \quad Z = \frac{z}{t}. \tag{1}$$

The parameters a_H and p_H correspond to the Hertz dry contact half-width and peak pressure, respectively. They are based on the mechanical properties of the elastomeric layer:

$$a_\mathrm{H} = \sqrt{\frac{8\overline{W}R}{\pi E'}}, \qquad p_\mathrm{H} = \frac{2\overline{W}}{\pi a_\mathrm{H}}. \tag{2}$$

As it is assumed that all elastic deformation occurs in the elastomeric layer, the effective elastic modulus reduces to:

$$\frac{2}{E'} = \frac{1-\nu^2}{E}. \tag{3}$$

The dimensionless scaling results in a fixed computational domain, irrespective of the process parameters, which offers the advantage of re-using numerical solutions in parameter sweeps for faster computation. The computational domain and corresponding mesh are shown in Figure 1. It has a dimensionless length of 20 and unit dimensionless height, due to the above definition of X and Z.

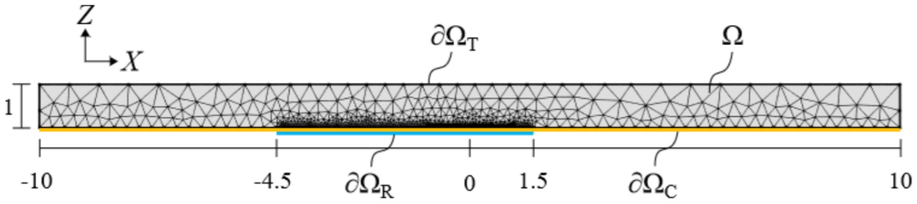

Figure 1: Dimensionless computational domain and mesh.

A.1 Elastic deformation

The elastic deformation is determined by applying the classical linear elasticity equations on the elastic layer domain Ω in Figure 1, with appropriate boundary conditions. The dimensionless linear elasticity equations are given by [1]:

$$\begin{aligned}
&X\text{-direction: } \frac{\partial}{\partial X}\left[(\lambda+2\mu)\frac{t}{a_\mathrm{H}}\frac{\partial U}{\partial X} + \lambda\frac{\partial W}{\partial Z}\right] + \frac{\partial}{\partial Z}\left[\mu\left(\frac{a_\mathrm{H}}{t}\frac{\partial U}{\partial Z} + \frac{\partial W}{\partial X}\right)\right] = 0,\\
&Z\text{-direction: } \frac{\partial}{\partial X}\left[\mu\left(\frac{\partial U}{\partial Z} + \frac{t}{a_\mathrm{H}}\frac{\partial W}{\partial X}\right)\right] + \frac{\partial}{\partial Z}\left[\lambda\frac{\partial U}{\partial X} + (\lambda+2\mu)\frac{a_\mathrm{H}}{t}\frac{\partial W}{\partial Z}\right] = 0,
\end{aligned} \tag{4}$$

where λ and μ correspond to the Lamé parameters:

$$\lambda = \frac{\nu E_{\text{eq}}}{(1-2\nu)(1+\nu)}, \qquad \mu = \frac{E_{\text{eq}}}{2(1+\nu)}. \tag{5}$$

and where E_{eq} corresponds to the equivalent elastic modulus of the elastic layer, which is defined as follows:

$$E_{\text{eq}} = E\frac{a_{\text{H}}}{R p_{\text{H}}}. \tag{6}$$

The elastic modulus is multiplied by a_{H}/R, to directly obtain the dimensionless displacement vector. Furthermore, it is divided by p_{H}, such that the dimensionless tensioned web contact pressure P_{C} can be used as a pressure load on domain boundary $\partial\Omega_{\text{C}}$ [1]. The upper boundary $\partial\Omega_T$ in Figure 1 is fixed.

A.2 Hydrodynamic lubrication

The dimensionless, steady-state, incompressible Reynolds equation in one dimension is given by:

$$\frac{\partial}{\partial X}\left(-\frac{a_{\text{H}}^3 p_{\text{H}} H^3}{12R^2\eta}\frac{\partial P}{\partial X} + \frac{H(u_1+u_2)}{2}\right) = 0. \tag{7}$$

The Reynolds equation is applied on domain $\partial\Omega_{\text{R}}$ in Figure 1, with zero pressure boundary conditions. The hydrodynamic film pressure P is determined for a given layer height profile H:

$$H(X) = H_0 + \frac{X^2}{2} + W(X), \tag{8}$$

where H_0 is the unknown dimensionless gap between the roller and substrate at $X = 0$. The second term is an approximation to describe the circular roller shape, and the last term represents the dimensionless elastic deformation, which follows from Equation (4).

A.3 Web tension

The dimensionless tensioned web contact pressure P_{C} is given by:

$$P_{\text{C}} = P + \frac{T}{R p_{\text{H}}}\left(1 + \frac{\partial^2 W}{\partial X^2}\right). \tag{9}$$

It is applied on domain $\partial\Omega_{\text{C}}$ in Figure 1, with zero pressure boundary conditions.

A.4 Load balance

The dimensionless load equilibrium is given by:

$$\int_{\Omega_{\text{C}}} P \mathrm{d}X = \frac{\pi}{2}. \tag{10}$$

This equation is satisfied by regulating the gap H_0 in Equation (8), which is one of the unknowns in the system of equations.

B Nomenclature

Dimensional

a_{H}	Hertz contact half-width (m)
E	Elastic modulus (Pa)
E'	Effective elastic modulus (Pa)
f	Liquid volume fraction (-)

Dimensionless

H	Film/layer height (-)
H_0	Gap between roller and substrate at $X = 0$ (-)
P	Hydrodynamic film pressure (-)
P_{C}	Tensioned web contact pressure (-)

h	Film/layer height (m)
h_0	Gap between roller and substrate at $x = 0$ (m)
h_C	Central layer height (m)
h_F	Final layer height (m)
h_M	Minimum layer height (m)
p	Hydrodynamic film pressure (Reynolds) (Pa)
p_C	Tensioned web contact pressure (Pa)
p_H	Hertz contact pressure (Pa)
p_n	Normal pressure on the tensioned web (Pa)
R	Roller radius (m)
T	Web tension ($\mathrm{N\,m^{-1}}$)
t	Elastomeric layer thickness (m)
u	Elastic deformation in x (m)
u_1	Roller surface imprint velocity ($\mathrm{m\,s^{-1}}$)
u_2	Substrate surface imprint velocity ($\mathrm{m\,s^{-1}}$)
w	Elastic deformation in z (m)
$\overline{W}$	Effective imprint load per unit length ($\mathrm{N\,m^{-1}}$)
x	Space coordinate in horizontal direction (m)
z	Space coordinate in vertical direction (m)
z_{roller}	Roller height profile (m)
η	Resin dynamic viscosity (Pa s)
θ	Cavity fraction $(1 - f)$ (-)
κ	Curvature of the tensioned web ($\mathrm{m^{-1}}$)
λ	Lamé's first parameter (Pa)
μ	Lamé's second parameter (Pa)
ν	Poisson ratio (-)
σ_n	Normal component of the stress tensor (Pa)
σ_t	Tangential component of the stress tensor (Pa)
U	Elastic deformation in X (-)
W	Elastic deformation in Z (-)
X	Space coordinate in horizontal direction (-)
Z	Space coordinate in vertical direction (-)

References

[1] W. Habchi, *Finite Element Modeling of Elastohydrodynamic Lubrication Problems*. Hoboken, NJ: John Wiley & Sons, 2018, ISBN: 978-1-119-22514-0 978-1-119-22515-7.
